# Seborrheic Pemphigoid

**DOI:** 10.1155/2014/768217

**Published:** 2014-08-18

**Authors:** Enzo Errichetti, Giuseppe Stinco, Enrico Pegolo, Nicola di Meo, Giusto Trevisan, Pasquale Patrone

**Affiliations:** ^1^Institute of Dermatology, Department of Experimental and Clinical Medicine, University of Udine, San Michele Hospital, Piazza Rodolone 1, Gemona del Friuli, 33013 Udine, Italy; ^2^Institute of Anatomic Pathology, Department of Medical and Biological Sciences, University of Udine, University Hospital of Santa Maria della Misericordia, Piazzale Santa Maria della Misericordia 15, 33100 Udine, Italy; ^3^Institute of Dermatology and Venereology, University of Trieste, Maggiore Hospital, Piazza Ospedale 1, 34100 Trieste, Italy

## Abstract

Seborrheic pemphigoid (SP), first described in 1969 by Schnyder, is a peculiar variant of BP which clinically resembles pemphigus erythematosus, since it is characterized by ruptured bullae and erosions covered with crusts involving the seborrheic areas. To the best of our knowledge, from the first description only four other cases of SP have been reported, of which two are in the English literature. We report an additional case of SP in a 56-year-old man with cervical spondylogenic myelopathy with very impaired mobility.

## 1. Introduction

Bullous pemphigoid (BP) is a chronic, autoimmune, often pruritic, subepidermal, blistering dermatosis occurring mainly in elderly individuals aged 70 years and older. Classically, patients present with large tense bullae on apparently normal or erythematous skin, located at the sides of the neck, axillae, groins, upper inner aspects of the thighs, and abdomen. Not rarely, excoriated, eczematous, papular, or urticarial lesions may precede the onset of the blisters [1, 2]. In addition to the more classic findings, several atypical presentations of BP have been described, including forms confined to a particular cutaneous district (paralyzed extremity, pretibial area, umbilicus, vulva, and irradiated or peristomal sites) and variants presenting with vesicular, erythroderma, vegetating, dyshidrotic dermatitis-like, prurigo nodularis-like, toxic epidermal necrolysis-like, ecthyma-like, or pemphigus erythematosus-like lesions [2, 3].

The latter variant is known as “seborrheic pemphigoid” and is a very rare form of BP, since there are only a few cases reported in the literature. We report an additional case of seborrheic pemphigoid in a patient with cervical spondylogenic myelopathy with very impaired mobility.

## 2. Case Report

**Figure 1:**
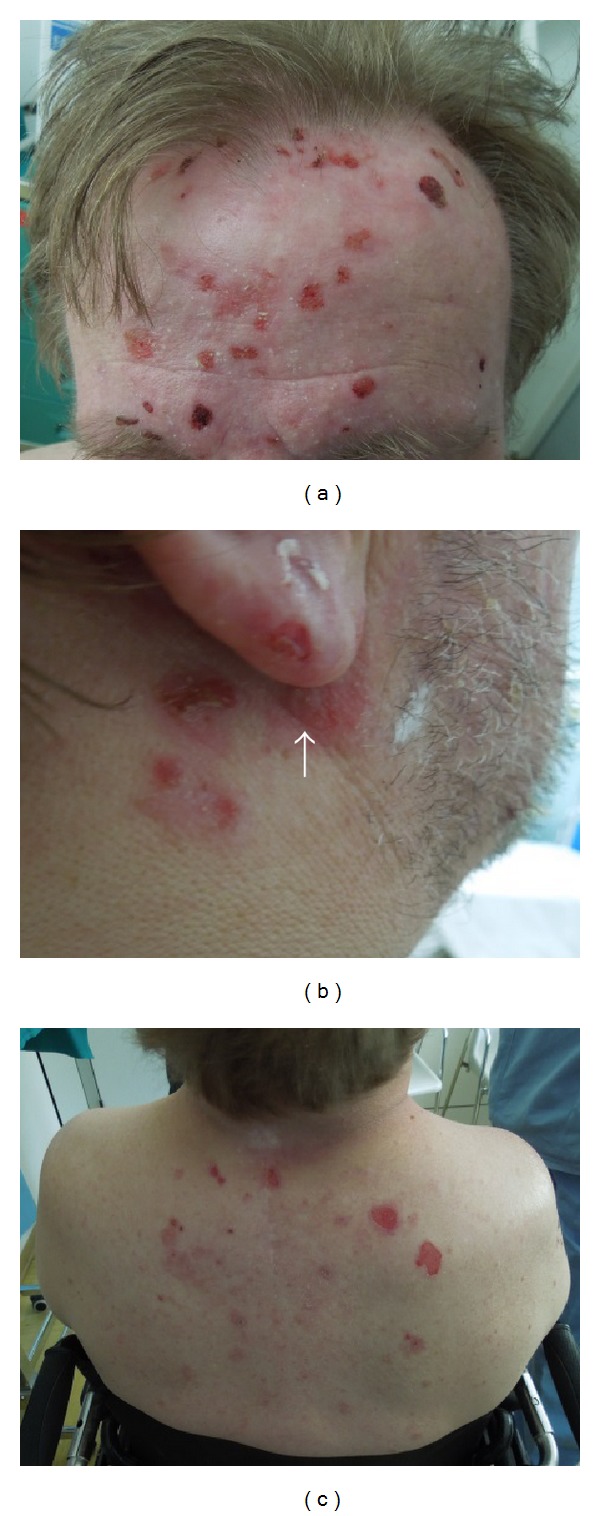
Several erosions (some of which with a peripheral epithelial collarette) and hematic and serous crusts on a slightly erythematous background on forehead (a), right periauricular region (b), and interscapular area (c); a flaccid blister (arrow) is visible under the right ear lobe (b).

**Figure 2:**
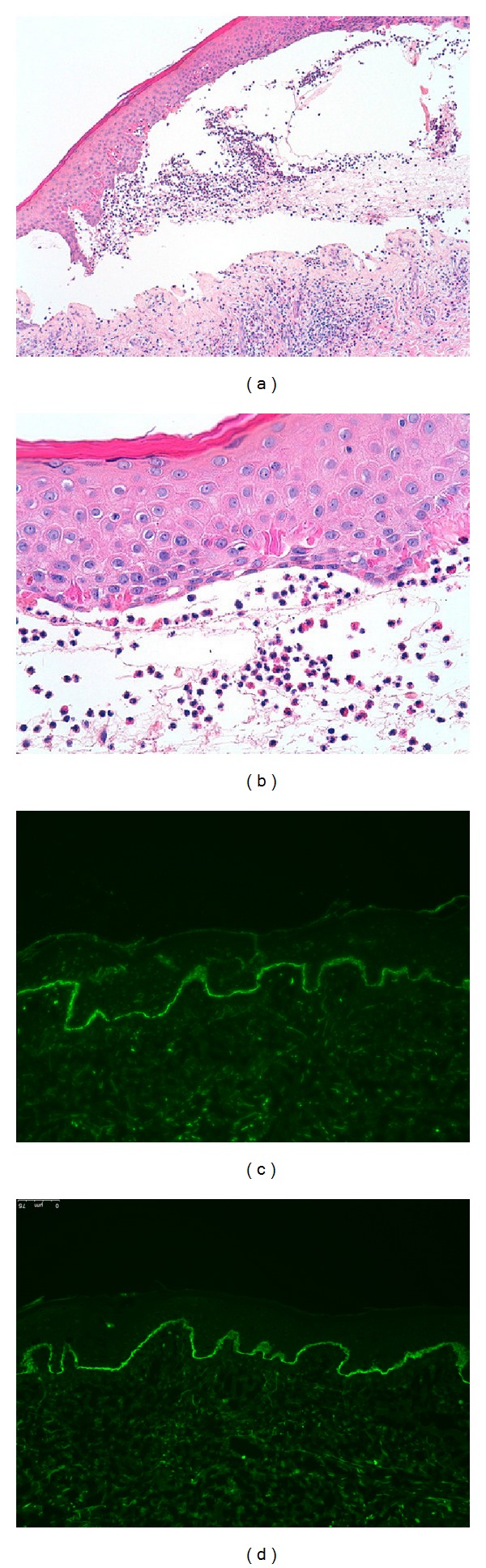
Subepidermal bulla containing eosinophils with an eosinophilic inflammatory cell infiltrate in the superficial dermis (H and E staining, magnification ×100) (a); detail of eosinophils in the subepidermal blister (H and E staining, magnification ×400) (b). Direct immunofluorescence tests show deposition of IgG (c) and C3 (d) at the basement membrane zone (200x).

A 56-year-old man presented to our clinic with a three-month history of recurrent and mild itchy bullae quickly evolving in erosive lesions covered by crusts located on his scalp, forehead, auricular and periauricular regions, and interscapular area (seborrheic sites). The patient had been previously diagnosed as having impetigo and seborrheic dermatitis; however, specific therapies for these disorders were found to be completely ineffective. His medical history included an untreated progressive (during previous 4 years) slight bilateral age-related sensorineural hearing loss (presbycusis); bipolar disorder (since he was 35 years old) controlled from about 5 years with quetiapine, duloxetine, and lorazepam; and cervical spondylogenic myelopathy with very limited mobility (from about 2 years) which had worsened considerably in the last six months. The cervical problem has been treated only with physical therapies (but the patient is currently waiting for a surgical treatment). The man denied other drugs intake and health issues. Physical examination revealed very few flaccid blisters, several erosions (some of which with a peripheral epithelial collarette), and hematic and serous crusts on a slightly erythematous background (Figures 1(a), 1(b), and 1(c)); Nikolsky's sign was absent. No other significant skin or mucosal lesions were seen. A 6 mm punch biopsy specimen was taken from the edge of a blister of right periauricular region and submitted for histological examination, which showed subepidermal bulla containing eosinophils with an eosinophilic inflammatory cell infiltrate in the superficial dermis (Figures 2(a) and 2(b)). Direct immunofluorescence (IF) of perilesional skin detected IgG and C3 deposition at the basement membrane zone (Figures 2(c) and 2(d)). The result of immunoblotting showed IgG autoantibodies which reacted against BP230 in epidermal extracts; furthermore, the BP180 antibodies were also detected (value of 31.7 U/mL; cutoff value for positivity: 15.0 U/mL) by an enzyme-linked immunosorbent assay (ELISA) BP180-NC16a diagnosis kit. The detection of antinuclear and antiextractable nuclear antigens antibodies was negative. On the basis of clinical, histological, and laboratory findings, a diagnosis of seborrheic pemphigoid was made. Treatment with oral methylprednisolone at the dosage of 0.5 mg/kg/die mg produced a rapid improvement. After 5 weeks the patient achieved a complete remission and the steroid was gradually tapered during the subsequent 6 months up to dose of 0.1 mg/kg/die. The value of circulating anti-BP180-NC16a antibodies (ELISA test) was also progressively decreased (26.4 U/mL after 5 weeks, 21.6 U/mL after 3 months, and 18.1 U/mL after 6 months). Currently, after 9 months from the start of steroid therapy, the patient is free of disease and presents a value of circulating anti-BP180-NC16a antibodies under the cutoff value for positivity (13.2 U/mL) with a dose of methylprednisolone of 0.1 mg/kg/die.

## 3. Discussion

Seborrheic pemphigoid (SP) is a peculiar variant of BP which clinically resembles pemphigus erythematosus (known also as seborrheic pemphigus), since it is characterized by ruptured bullae and erosions covered with crusts involving the seborrheic areas [4]. The first instance of SP reported in the literature dates back to 1969, when Schnyder described a case in an elderly female [5]. It is important to underline that all cases [6–16] described as “SP” before Schnyder's report were actually instances of pemphigus erythematosus [5]. Such confusion was probably caused by the lack of availability of reliable serological tests and direct IF test of perilesional skin, which are very helpful particularly in the cases without detectable bullae [1, 17, 18]. To the best of our knowledge, since the first description four other instances of SP, similar to our case and original report of Schnyder, have been reported [1, 4, 17, 18], of which two are in English language in 1991 [18] and 2002 [1], respectively. Regarding the latter report, the authors emphasized a possible association between losartan intake and unleashing of the lesions in their patient [1].

When a blister is available, its histological examination shows a picture comparable to the classic form of BP, with a subepidermal cleavage [1]. Direct IF test of perilesional skin, which generally reveals linear deposition of IgG and C3 at the dermoepidermal junction [1], and detection of circulating IgG antibodies against basement membrane zone (indirect IF), BP230, and/or BP180 may be of aid in the diagnosis [1, 18].

The reasons underlying the peculiar location of the lesions in seborrheic areas in SP are not clear. It is well-known that the regional variability in the BP antigens skin expression may play a role in the distribution of the lesions of BP, given that the greatest concentration of BP antigens is in the skin of flexor surfaces of the arm, leg, and thigh, the most common sites involved in BP [19]. On the basis of this finding, it is possible to speculate that subjects with SP could present a higher expression of BP antigens in the seborrheic areas than the rest of the skin surface. Unfortunately, our patient denied further skin biopsies and therefore we have not been able to assess this hypothesis. We hope that future studies may evaluate this assumption. Another possible explanation for the peculiar localization of the lesion in SP could be that unknown factors typical of seborrheic areas may trigger or aggravate the disease in susceptible individuals. In this view, it is important to underline the ability of* Malassezia* yeast, which is notoriously localized to seborrheic sites, to activate the complement system, via either the alternative pathway or the classical pathway, with the possibility to amplify the complement-mediated inflammation which is characteristic of BP [20]. Considering that an increase of the static pool of already secreted sebum due to immobility and muscular paralysis plays a permissive role for growth of* Malassezia* yeast [21], the possible involvement of this microorganism could also explain a possible correlation between SP and cervical spondylogenic myelopathy with very limited mobility in our patients. In fact, albeit we can not exclude a coincidental association, there are three points that support a such link: the development of the disease after a relatively brief period from the significant worsening of the motor abilities, the age of onset lower than the average [3], and the well-known correlation between classical pemphigoid and neurodegenerative processes [22, 23].

Although SP may sometimes be confused with seborrheic dermatitis or impetigo, the main differential diagnosis is pemphigus erythematosus. In our opinion, the negativity of Nikolsky's sign, as described in the present case, may help to suspect a SP rather than pemphigus erythematosus, since in the latter it is almost always present [24]. Anyhow, only the histology, direct immunofluorescence examination, and serological studies allow us to definitely distinguish SP from pemphigus erythematosus and the other conditions mentioned above.

With regard to the therapy, according to other authors [3], our case confirms that relatively low dosages of systemic corticosteroids are effective in SP.
